# Characteristics and Effectiveness of XP-Endo Files and Systems: A Narrative Review

**DOI:** 10.1155/ijod/9412427

**Published:** 2024-12-17

**Authors:** Sarah M. Alkahtany, Rana Alfadhel, Aseel AlOmair, Sarah Bin Durayhim

**Affiliations:** ^1^Department of Restorative Dental Sciences, College of Dentistry, King Saud University, P. O. Box 68004, Riyadh 11527, Saudi Arabia; ^2^College of Dentistry, King Saud University, P. O. Box 68004, Riyadh 11527, Saudi Arabia

## Abstract

**Background:** XP-endo files are composed of Max-Wire alloy, which was developed by FKG Dentaire (La Chaux-de-Fonds, Switzerland). This alloy, known as Martensite-Austenite Electropolish Flex, is the first NiTi alloy used in endodontics to combine the shape memory effect with superelasticity for use in clinical practice.

**Objective:** This article aims to provide a comprehensive overview of the existing knowledge and evidence regarding different XP-endo files and systems, XP-endo Finisher (XPEF), XP-endo Shaper (XPES), XP-endo Retreatment (XPER), XP-endo Rise (XPE-Rise), and XPE-Rise Retreatment (XPE-Rise-R), to help clinicians understand their different properties and recommended clinical uses. Moreover, this review aims to identify future research opportunities in this field.

**Materials and Methods:** A search was performed in the PubMed database using the keywords “XP endo” or “XP-endo”. A total of 309 studies were identified during the initial search, and then initial abstract screening was conducted. The inclusion criteria included any study that aimed to evaluate XP endo files and systems on permanent teeth, either in vivo or in vitro. We excluded studies that were unrelated to the subject, literature reviews, case studies, and studies that employed deciduous teeth. Finally, we performed an extensive review of the selected 130 studies, which we assessed, summarized, and classified based on the specific XP-endo file used.

**Conclusion:** Overall, XP-endo files offer significant improvements in endodontic treatment. XPEF excels in irrigation activation and medicament removal, and XPES succeeds in canal cleaning, shaping, and retreatment. The XPER system, which incorporates the XPEF-R file, shows promise in removing root-filling materials, but its performance is inconsistent compared to other retreatment systems. We have found limited information regarding the latest XPE-Rise and XPE-Rise-R systems, additional research is required to fully determine their comparative effectiveness and optimize their clinical application.

## 1. Introduction

Endodontists are continually exploring different instruments and techniques to optimize the cleaning and shaping of the root canal system. However, none of the existing systems are capable of making full contact with all the walls of the root canal. Due to the complicated configuration and morphology of root canals, there are always untouched regions within the prepared dentin [1, 2]. Manufacturers have invested considerable effort in developing an endodontic file that possesses unique three-dimensional shaping capabilities. They produce these innovative NiTi files by combining modified alloys with various treatments and innovative file designs. Scientists conducted comprehensive investigations using a variety of laboratory and clinical testing methods to thoroughly evaluate the effectiveness of the new NiTi endodontic files. They examined their cutting efficiency, shaping ability, cleaning, and disinfection efficiency, root canal filling removal ability, and fracture resistance [3].

All XP-endo files are composed of Max-Wire alloy, which was developed by FKG Dentaire (La Chaux-de-Fonds, Switzerland). This alloy, known as Martensite-Austenite Electropolish Flex, is the first NiTi alloy used in endodontics to combine the shape memory effect with superelasticity for use in clinical practice [3]. The manufacturer first introduced the XP-endo Finisher (XPEF) file in 2015 as a supplementary tool to be used after cleaning and shaping with any other system. In 2016, the XP-endo Shaper (XPES) was introduced as a disposable single-use file designed for canal cleaning and shaping. In 2017, the company launched the XP-endo Retreatment (XPER) set, including the Shaper and the Finisher R; this set was used for nonsurgical retreatment. The most recent systems, the XP-Endo Rise (XPE-Rise) and the XPE-Rise Retreatment (XPE-Rise-R) were introduced in 2022 and featured a redesigned tip. Different abbreviations used for each XP-endo file and system were defined in Table 1.  Table 2 summarizes the design, components, uses, recommended speed, and torque for various XP-endo files and systems.

This review article aims to provide a comprehensive overview of the existing knowledge and evidence regarding the characteristics and effectiveness of different XP-endo files (XPEF, XPES, XPER, XPE-Rise, and XPE-Rise-R) to help clinicians understand their different properties and recommended clinical uses. Moreover, this review aims to identify future research opportunities in this field (Table 1).

## 2. Materials and Methods

A search was performed in the PubMed database from 2016 until September 2024, using the keywords “XP endo” or “XP-endo.” A total of 309 studies were identified during the initial search, and then initial abstract screening was conducted. The inclusion criteria included any study that aimed to evaluate XP-endo files and systems on permanent teeth, either in vivo or in vitro. We excluded studies that were unrelated to the subject, narrative literature reviews, case studies, and studies that employed deciduous teeth. Systematic reviews and meta-analyses were included. Finally, we performed an extensive review of the selected 125 studies, which we assessed, summarized, and classified based on the specific XP-endo file used.

This review paper included four sections discussing the main XP-endo files and systems: XPEF, XPES, XPER, and XPE-Rise. In each section, we provided a concise overview of the file's design and the manufacturer's recommendations. Furthermore, we presented the file's applications, effectiveness, and characteristics based on the reviewed studies.

### 2.1. XPEF

#### 2.1.1. Characteristics and Physical Properties

**Figure 1:**
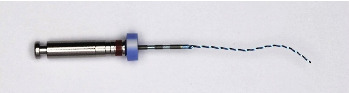
XP-endo Finsiher (XPEF).

The XPEF (FKG Dentaire, La Chaux-de-Fonds, Switzerland) is a nontapered, small-core, hook-like rotary file made of MaxWire alloy (Figure 1). It was originally introduced for use as a supplementary file after root canal preparation; it would serve as a final step to improve root canal cleaning while conserving dentin. It was designed to remove intracanal medicaments and root-filling materials. This file has enhanced cyclic fatigue resistance [4].

The manufacturer recommends its use only after a minimum preparation of ISO size 25. Then, XPEF is implemented for 1 min at a speed of 800–1000 rpm and a torque of 1 Ncm (Table 1). This file is supplied inside a plastic tube. The working length should be measured before removing the tube, and cold spray should be applied to keep the XPEF straight (in the martensitic phase) for easier insertion into the canal. When this file is inserted into the canal and exposed to body temperature, a phase transformation occurs, and the file's shape becomes more curled (in the austenitic phase). The shape transformation of XPEF will occur at its optimal temperature of 33.9 ± 1.4°C [5]. The irrigation solution reached a maximum temperature of 35.5°C after 211 s of XPEF agitation [6].

#### 2.1.2. Irrigation Activation and Cleaning Efficiency

Manufacturers have recommended using XPEF to activate the irrigating solution used in root canal procedures, which improves the cleaning efficiency of the file system used for chemo-mechanical preparation [7, 8]. Previous studies have demonstrated that XPEF can effectively remove accumulated hard tissue debris and smear layers from the root canal system. However, researchers have reported contradictory findings when comparing XPEF to other irrigation activation systems. Some studies found that this supplementary file improved irrigation cleaning efficiency throughout the length of the canal compared to other techniques, such as the EndoVac, the EndoActivator, passive ultrasonic irrigation (PUI), and standard needle irrigation [9–12]. Other investigations found that XPEF was as effective as needle irrigation, PUI, the EndoActivator, and the Endovac in its ability to remove hard tissue debris [13–17]. On the contrary, scanning electron microscopic (SEM) findings showed that XPEF was inferior to PUI in smear layer removal [18].

#### 2.1.3. Bacterial Reduction

The antibacterial action of the irrigation will be enhanced by the use of XPEF; bacterial reduction will be extended 50 µm into the dentinal tubules [19]. However, no irrigation activation method can disinfect the root canal space completely. A previous investigation found that the supplementary use of XPEF will reduce the bacteria better than Endoactivator, Endoclean, and PUI [20]. Furthermore, biofilm removal was significantly improved by using XPEF [21, 22]. Activation of 1.5% sodium hypochlorite (NaOCl) with XPEF will be effective against *Fusobacterium*, *Actinomyces*, *Porphyromonas*, *and Capnocytophaga*, but it is not effective against *E. faecalis* [23–25]. The antibacterial effect against *E. faecalis* would be better if 2.5% NaOCl was activated with XPEF or EndoActivator during retreatment procedures [26]. The XPEF activation or any other irrigation activation method will only significantly affect the bacteria in the main canal, not the bacteria in the lateral canals [27].

On the other hand, another study reported that XPEF's disinfective ability was comparable or even less to that of PUI [28–30]. A systematic review conducted by de Olivera found no evidence to support the use of XPEF for bacterial reduction in the root canal system [31]. In a randomized clinical trial, the supplementary use of XPEF after a reciprocating file did not improve the outcome of root canal treatments [32].

#### 2.1.4. Removal of Intracanal Medicaments

XPEF enhanced the removal of calcium hydroxide (Ca[OH]_2_) paste from the root canal system [33–38]. It was found to be superior to PUI in the apical third after 3 min of activation [39]. A study using confocal laser scanning microscopy revealed that the use of XPEF during irrigation activation enhances the penetration of the endodontic sealer into dentinal tubules, as compared to conventional needle irrigation when removing Ca(OH)_2_ [33]. Moreover, XPEF removed significantly more double and triple antibiotic paste from the straight immature root canals than needle irrigation and PUI [40, 41]. These findings were contrary to those of a systematic review by Zhou, Liu, and Guo [42], who reported improved medicament removal by PUI compared to XPEF. Moreover, PUI will improve the irrigant tubular penetration during the removal of Ca(OH)_2_ more effectively than XPEF in the apical third [43].

#### 2.1.5. Retreatment Procedures

Previous investigators tested XPEF's ability to remove root-filling material following the use of other systems, although this file was not designed for retreatment. The results revealed that XPEF can enhance the removal of filling materials from root canals after using the ProTaper Universal Retreatment (PTUR) and WaveOne Gold (WOG) systems [44, 45]. Furthermore, it can enhance root filling removal following the use of Reciproc retreatment files, but it cannot remove the filling completely [46]. A recent micro-CT analysis revealed that XPEF outperformed the EndoActivator, PUI, and ProTaper universal systems in removing BC sealers from the root canals [47]. However, XPEF should be used with caution during retreatment procedures because it might fracture without any signs of deformation. Most fractures that occur (81%) in XPEF are due to cyclic fatigue [5].

#### 2.1.6. Irrigation Extrusion, Postoperative Pain, and Cracks Creation

XPEF was associated with a higher chance of irrigation extrusion compared to the standard needle irrigation and other activation systems, especially in immature roots [48–51]. However, if used appropriately, it will not cause postoperative pain following endodontic treatment [52, 53]. Irrigation activation with XPEF was found to cause more pain than diode laser and PUI activation. However, this pain will disappear completely after 2 weeks [54]. Postoperative pain levels were higher in cases of overinstrumentation that led to a widening of the apical foramen [55]. Furthermore, XPEF did not induce any new cracks in previously prepared canals [56].

#### 2.1.7. Regeneration

Azimian et al. [13] suggested that ethylenediaminetetraacetic acid (EDTA) activated with XPEF could be useful in certain clinical situations—such as the second visit of a regenerative endodontic procedure—in which NaOCl cannot be used due to its negative impact on stem cells. Their SEM study compared the debris and smear layer removal of the standard needle irrigation technique, which uses EDTA and NaOCl, to that of XPEF's activation of EDTA solution. The resultant SEM images were similar for the two techniques. Moreover, XPEF will enhance the action of EDTA to release more growth factors from dentin that are required for successful tissue regeneration [57].

### 2.2. XPES

#### 2.2.1. Characteristics and Physical Properties

**Figure 2:**
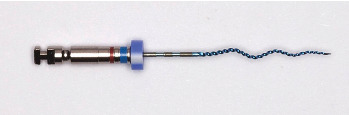
XP-endo Shaper (XPES).

XPES is a continuously rotating, single-use, snake-like file with a triangular cross-section made by FKG Dentaire SA (La Chaux-de-Fonds, Switzerland). Although it is constructed with the same MaxWire alloy as XPEF, its usage and design are entirely different. The file has a booster tip consisting of six blades, which enables it to navigate and shape the canal following a manual glide path of at least ISO size 15 (Figure 2). It then gradually enlarges the apical preparation size up to ISO size 30, with 0.04 canal flaring [58, 59]. A previous investigation tested the XPES if used multiple times; after the second use, microcracks were evident, then they propagated, resulting in file fracture after the fourth use [60].

The manufacturer's recommendation is to use the XPES file at a rotational speed of 1000 rpm and a torque of 1 Ncm. A prior investigation indicated that it can be used without risk, even when operating at a speed of 3000 rpm [60].

The MaxWire alloy and unique design—with a narrow core diameter and minimal initial taper—have contributed to the broad flexibility of XPES. The file begins in the martensitic phase. Upon insertion into the canal and exposure to body temperature, the file transitions into the austenitic phase. As a result, the file's geometry changes according to its memory to assume a spiral shape, and the taper increases to 0.04 [61–63]. XPES will have the ideal shape transformation at 35.1 ± 1.0°C [5]. The MaxWire alloy and small initial taper of XPES account for its high cyclic fatigue resistance compared to other instruments, such as the K3XF, ProTaper Gold (PTG), TRUShape, HyFlex CM (HCM), FlexMaster, Vortex Blue and iRace systems [46, 59, 64–66]. However, the torsional fatigue resistance of XPES was found to be inferior to other tools, such as the TRUShape, Profile Vortex, and FlexMaster systems, due to its smaller cross-sectional area [66, 67]. A new finite element analysis study found that the XPES caused minimal stresses on the root canal dentinal walls compared to the TruNatomy, F360, and 2-shape systems. However, the difference was not significant [68].

#### 2.2.2. Cleaning and Shaping Ability

According to microcomputed tomography (microCT) studies, XPES performed well in cleaning and shaping root canals but, like any other system, left certain areas of the dentinal walls untouched [69]. However, some investigations have reported that XPES was associated with a decrease in untouched root canal surfaces; at the same time, it was more conservative, decreased the amount of canal transportation, and altered the root canal anatomy less compared to the ProTaper universal (PTU), BioRace, Reciproc Blue (RB), Reciproc systems, Self-Adjusted File (SAF), Hyflex, EDGEFILE X-3, TRUNATOMY, WOG, and TRUShape rotary files, as well as the ProTaper Next (PTN) [70–84]. If the time of instrumentation with XPES were extended for an extra 45 s, its shaping ability would be similar to that of PTN [70]. Wang et al. suggested employing XPES in 10 picking motions at the working length for each canal to enhance its cutting efficiency and minimize the untouched wall areas while keeping the canal's morphological features unchanged [85, 86].

In contrast, recent micro-CT and CBCT investigations reported that XPES was similar to WOG in canal centralization ability, and XPES was associated with more transportation compared to the TruNatomy and HCM file systems at 5 mm level [71, 72]. Another study reported less transportation with the use of XPES compared to RB and WOG [87]. The supplementary use of XPEF after XPES will significantly reduce the untouched dentinal wall areas [73].

According to a previous study, XPES produces the least amount of debris and smear layer in comparison to PTU and Twisted File (TF) [88]. XPES demonstrated superior cleaning efficacy, improved instrumentation, and a reduced number of untouched areas compared to the Mtwo system in oval canals. Nevertheless, they possess comparable capabilities for centering and exhibit comparable incidences of transportation [89–91].

XPES was successfully used in C-shaped canals in previous publications [83]. XPES left the walls 43% untouched after instrumentation of a C-shaped canal configuration, which was comparable to the performance of BioRace, TRUShape, and RB. However, XPES facilitates less accumulation of hard tissue debris in C-shaped canals [84, 92]. All existing systems leave undetectable bacteria in half of the tested samples [85].

#### 2.2.3. Bacterial Reduction

Microbiological studies have indicated that no instrumentation system or technique renders root canals completely free of bacteria. XPES significantly decreased the bacterial load in root canals and reduced significantly more bacteria than the WOG, PTG, PTN, HCM, and TruNatomy. However, its performance was similar to that of the Hyflex EDM, iRaCe, and RB files. A significantly greater reduction in bacteria load was found when XPEF was used after XPES [74–81].

#### 2.2.4. Retreatment Procedures

XPES has the capability to remove the core filling material; however, it lacks the ability to completely eliminate the sealer [93]. Ciftcioglu et al. utilized XPES for BC sealer retreatment. Their study found that it was associated with a lower amount of debris extrusion in comparison to PTUR and RB [94]. Moreover, a study showed that XPES had greater efficacy in BC sealer retreatment compared to R-Endo files [95, 96].

#### 2.2.5. Debris Extrusion, Postoperative Pain, Crack Formations

Less apically extruded debris was created by XPES than by RB, WOG, TF, PTG, HPT, and the Hero Shaper [61, 82, 87]. Furthermore, XPES was associated with less postoperative pain and flare-up than iRaCe files [76].

The Xavier et al. [91] randomized controlled trial revealed that patients who received XPES system instrumentation experienced a higher level of postoperative pain compared to those who received WOG instrumentation. Additional research has demonstrated that the use of the XPES system resulted in lower levels of postoperative pain compared to other instruments such as PTU, PTG, HyFlex EDM OneFile, WOG, 2shape, and RB [93–95].

According to the manufacturer, XPES minimizes the risk of dentin microcracks by applying minimum stress to dentinal walls [86]. In micro-CT studies, none of the tested systems—PTG, SAF, WOG, RB, and XPES—induced new dentinal microcracks. However, using the PTU and PTUR systems to clean and shape the canal significantly increased the proportion of microcracks [97–99]. The results were different in our previous micro-CT investigation, which revealed more microcracks in the middle third of the root when XPES instrumentation was used. We explained this finding on the basis of the high rotational speed required for the XPES system and the nature of its movement, which was associated with greater vibration than that created by the PTU system [88]. A previous microscopic evaluation found that the use of XPES was associated with less crack formation compared to TruNatomy, RB file, Neoendoflex file, and Hero Shaper file systems [89, 90].

### 2.3. XPER

#### 2.3.1. Characteristics and Physical Properties

**Figure 3:**
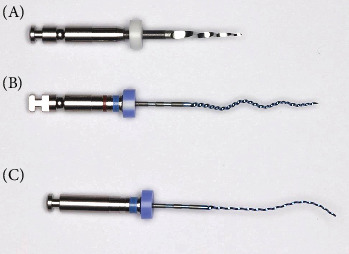
XP-endo Retreatment system (XPER). DR1 (A), XP-endo Shaper (B), and XP-endo Finisher-R (C).

The XPER system was put on the market for use in nonsurgical retreatment. This system combines three different files from sequences from FKG Dentaire's previous files, DR1 (from the D-Race system), XPES, and XPEF-R (Figure 3).

DR1 is the first file to be used in the XPER system. The same desobturation file used in the D-Race sequence, it is used to remove different root-filling materials from the coronal third. It has an active tip and 0.1 taper and is ISO size 30. The recommended speed is 1000 rpm, with a torque of 1.5 Ncm. Subsequently, XPES—the single-use shaping file described above—is used deeper into the canal. The recommended speed for retreatment cases is 1000–2500 rpm, with a torque of 1 Ncm.

Finally, XPEF-R is used to finish the root-filling retrieval phase of endodontic retreatment. The recommended speed is 800–1000 rpm, with a torque of 1 Ncm. XPEF-R will achieve the ideal shape transformation at 32.7 ± 1.7°C [5].

Liu et al. [100] conducted an SEM evaluation of clinically fractured XPES files. Their results revealed that 67% of fractures in XPES files used for retreatment were due to torsional fatigue; the file will exhibit plastic deformation as a warning sign before it fractures. The low torsional fatigue resistance is mainly due to the small core diameter. Torsional stresses will be higher due to the file's engagement with the root canal filling material during the retreatment procedure.

#### 2.3.2. Retreatment Procedures

Previous studies have failed to find any system capable of removing all residual filling material from root canals [46, 101–104]. The efficiency of the XPER system in cleaning out roots filled with gutta-percha (GP) and AH plus sealer was evaluated and compared to that of other retreatment files and techniques. It was found that XPES and XPER removed higher percentages of root-filling material compared to manual retreatment with an H-file and other rotary systems, such as PTUR, Reciproc, RB, and TRUShape. This removal efficiency could be improved by enlarging the apical preparation size [105–108].

#### 2.3.3. Supplementary Uses of XPEF-R With Other Retreatment Systems

A meta-analysis concluded that XPEF-R can be effectively used to remove root filling; however, the evidence was inconsistent [109]. The removal of root filling material was improved significantly when XPEF-R was used after canal retreatment as a supplement to systems such as Reciproc files [101, 110–112], PTUR [113], WaveOne [114], and D-Race [104]. The supplementary step with the XPEF-R instrument was associated with a 38% increase in the removal of filling material [107]. According to a micro-CT analysis, the supplementary use of XPEF-R after Mtow-R was most effective in the apical third; however, the difference was not significant [115]. A systematic review conducted by Yang et al. [116] concluded that there was no significant difference in the removal of remaining filling material between XPEF-R and ultrasonic activation. XPER system can remove GuttaCore and Thermafil GP [117]. A confocal scanning microscopic study reported that the supplementary use of XPEF-R during retreatment will not enhance the NaOCl dentinal tubule penetration [118].

#### 2.3.4. XPEF-R vs. XPEF

XPEF-R has a larger diameter than XPEF. Therefore, the Finisher R must be used only after preparing the canal to at least size ISO 30 and not size 25, as with XPEF. Furthermore, XPEF-R is a little stiffer, and its tip angle enhances its effectiveness in removing root-filling material attached to the walls of the canal, particularly in anatomically complex areas [46].

A stereomicroscopic study showed that XPEF-R cleaned root canals more effectively than XPEF when used as a supplementary file [103]. However, a different study found no significant difference between the finishers [46]. Agarwal et al. found that supplementary use of XPEF during the retreatment procedure resulted in better cleaning efficacy and less debris extrusion compared to XPEF-R [119, 120].

XPEF-R was found to be more effective in removing root-filling material (GP + AH Plus with a single-cone technique) than PUI, a Clearsonic tip, and the EndoActivator [101, 121, 122]. However, Crozeta et al. [102] reported that PUI instruments were more effective than the supplementary use of XPEF-R (following R50) in removing root-filling material when BC sealer was used.

#### 2.3.5. Bioceramic Sealer Retreatment

Removing BC sealer remains a significant challenge in endodontic retreatment. An analysis using microCT showed that the XPER system was successful in removing root-filling material consisting of GP and bioceramic sealer using both single cone and warm vertical techniques [123]. However, there is still a concern regarding the removal of the filling material from canals filled using the warm vertical condensation method in the critical apical area.

An SEM study showed that the cleaning efficiency of the complementary use of both XPEF and XPEF-R was superior to that of PUI after the retreatment of conventional and hydraulic calcium silicate sealer [124, 125]. The utilization of XPEF-R resulted in a faster establishment of canal patency [126].

### 2.4. XPE-Rise

#### 2.4.1. Characteristics and Physical Properties

The XPE-Rise, which emerged in 2022, represents the newest generation introduced by FKG. It contains two systems: one for initial treatment (XPE-Rise) and the other for retreatment (XPE-Rise-R).

**Figure 4:**
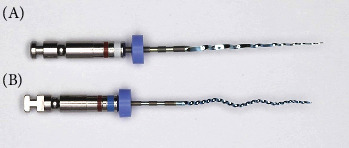
XP-endo Rise system (XPE-Rise). It is composed of XPE-Rise Glider (A) and XPE-Rise Shaper (B).

XPE-Rise is composed of two types of files: the XPE-Rise Glider and the XPE-Rise Shaper (Figure 4). The XPE-Rise Glider has a diameter of ISO 15 and a taper of 0.04. The XPE-Rise Shaper has a diameter of ISO 30 and a taper of 0.04. The manufacturer-recommended torque and speed are 1000 rpm and 1 Ncm, respectively.

**Figure 5:**
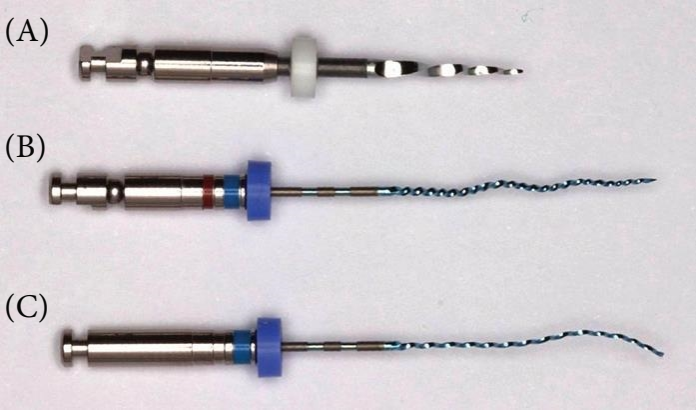
XP-endo Rise Retreatment system (XPE-Rise-R). DR1 (A), XP-endo Rise shaper (B), and XP-endo Finisher-R (C).

XPE-Rise-R system comprises three files: DR1, the XPE-Rise Shaper, and the XPEF-R (Figure 5). It is similar to the XPER system, which has the same files, except that the XPES is replaced by the XPE-Rise Shaper. The Shaper is recommended for use at 1000–2500 rpm and 1 Ncm.

#### 2.4.2. The Novelty of XPE-Rise

The XPE-Rise Shaper has a modified tip with six facets instead of three. According to the manufacturer, this increases predictability and control. It exhibits superior resistance to cyclic fatigue and superior flexibility in a shorter amount of time.

A comprehensive literature review was undertaken to evaluate the XPE-Rise. Only one study investigated the effects of various systems, including the XPE-Rise, on the stress exerted on the canal wall. The findings demonstrated that the XPE-Rise generated moderate stress in the middle and apical thirds of the canal. This level of stress was similar to that produced by XPES and higher than that induced by the iRace and Race EVO systems, which exerted low stress on the canal walls [127]. This is because XPE-Rise and XPES are both single-file systems. There is potential for future research on the other properties and characteristics of XPE-Rise.

## 3. Conclusion

Overall, XP-endo files offer significant improvements in endodontic treatment. XPEF excels in irrigation activation and medicament removal, but studies showed mixed results compared to other systems in its bacterial reduction and cleaning efficiency. The evidence supports the efficacy of XPES in canal cleaning, shaping, and retreatment, though some studies suggest it may induce more root canal transportation and microcracks under specific conditions. The XPER system, which incorporates the XPEF-R file, shows promise in removing root-filling materials, but its performance is inconsistent compared to other retreatment systems. We have found limited information regarding the latest XPE-Rise and XPE-Rise-R systems. The system provides improved designs with enhanced features. However, additional research is required to fully determine their comparative effectiveness and optimize their clinical application.

## Figures and Tables

**Table 1:** Different abbreviations used in this review.

Abbreviation	Definition
XPEF	XP-endo Finisher
XPES	XP-endo Shaper
XPER	XP-endo Retreatment System
XPEF-R	XP-endo Finisher Retreatment
XPE-Rise	XP-endo Rise system
XPE-Rise-S	XP-endo Rise shaper
XPE-Rise-G	Xp-endo Rise glidder
XPE-Rise-R	XP-endo Rise Retreatment system

**Table 2:** Summary of the design, components, uses, recommended speed, and torque for different XP-endo files and systems.

File system	Design and components	Uses	Recommended speed (RPM)	Recommended torque (Ncm)
XP-endo Finisher	Curved tip Hook-like file	• After root canal preparation to size 25 • Irrigation activation • C-shaped canal • Medicament removal • Retreatment	800–1000	1

XP-endo Shaper	Spiral shape Snake-like	• After glidepath of at least size 15 • Cleaning and shaping	1000	1

XP-endo Retreatment	DR 1 XPES XPEF-R (Hook-like)	Retreatment	1000 1000–2500 800–1000	1.5 1 1

XP-endo Rise	XPE-Rise-G XPE-Rise-S	Cleaning and shaping	1000	1

XP-endo Rise Retreatment	DR 1 XPE-Rise-S XPEF-R	Retreatment	1000 1000–2500 800–1000	1.5 1 1

## Data Availability

Not applicable to this review article as no new data were created or analyzed in this study.
